# Chemokine Receptors, CXCR1 and CXCR2, Differentially Regulate Exosome Release in Hepatocytes

**DOI:** 10.1371/journal.pone.0161443

**Published:** 2016-08-23

**Authors:** Hiroyuki Nojima, Takanori Konishi, Christopher M. Freeman, Rebecca M. Schuster, Lukasz Japtok, Burkhard Kleuser, Michael J. Edwards, Erich Gulbins, Alex B. Lentsch

**Affiliations:** 1 Department of Surgery, University of Cincinnati, College of Medicine, Cincinnati, Ohio, United States of America; 2 Institute of Nutritional Science, University of Potsdam, Nuthetal, Germany; 3 Department of Molecular Biology, University of Duisburg-Essen, Essen, Germany; Montana State University Bozeman, UNITED STATES

## Abstract

Exosomes are small membrane vesicles released by different cell types, including hepatocytes, that play important roles in intercellular communication. We have previously demonstrated that hepatocyte-derived exosomes contain the synthetic machinery to form sphingosine-1-phosphate (S1P) in target hepatocytes resulting in proliferation and liver regeneration after ischemia/reperfusion (I/R) injury. We also demonstrated that the chemokine receptors, CXCR1 and CXCR2, regulate liver recovery and regeneration after I/R injury. In the current study, we sought to determine if the regulatory effects of CXCR1 and CXCR2 on liver recovery and regeneration might occur via altered release of hepatocyte exosomes. We found that hepatocyte release of exosomes was dependent upon CXCR1 and CXCR2. CXCR1-deficient hepatocytes produced fewer exosomes, whereas CXCR2-deficient hepatocytes produced more exosomes compared to their wild-type controls. In CXCR2-deficient hepatocytes, there was increased activity of neutral sphingomyelinase (Nsm) and intracellular ceramide. CXCR1-deficient hepatocytes had no alterations in Nsm activity or ceramide production. Interestingly, exosomes from CXCR1-deficient hepatocytes had no effect on hepatocyte proliferation, due to a lack of neutral ceramidase and sphingosine kinase. The data demonstrate that CXCR1 and CXCR2 regulate hepatocyte exosome release. The mechanism utilized by CXCR1 remains elusive, but CXCR2 appears to modulate Nsm activity and resultant production of ceramide to control exosome release. CXCR1 is required for packaging of enzymes into exosomes that mediate their hepatocyte proliferative effect.

## Introduction

Hepatic ischemia/reperfusion (I/R) injury is a major cause of liver injury and dysfunction after extended liver resection, transplantation, and hemorrhagic shock [1–3]. The process of liver recovery and regeneration following hepatic I/R injury involves interactions between several cytokines, growth factors, and metabolic pathways to stimulate hepatocytes to drive cell cycles, replicate, restore liver mass [4–6]. CXC chemokines are known to play important roles in both liver injury and in the reparative and regenerative response to I/R [7–10]. Our previous work has demonstrated that the chemokine receptors, CXCR1 and CXCR2, expressed on hepatocytes, regulate liver recovery and regeneration after I/R injury in a manner directly related to ligand concentration in the hepatic microenvironment [11, 12].

More recently, we discovered that hepatocytes release exosomes during the course of I/R injury [13]. Exosomes are membrane nanovesicles (30–100 nm) released by cells into the extracellular environment upon fusion of multivesicular bodies with the plasma membrane [14–16]. Exosomes contain membrane components but also contain proteins, microRNAs and mRNAs [17, 18]. Our recent study demonstrated that hepatocyte-derived exosomes contain the synthetic machinery to form sphingosine-1-phosphate (S1P) in target hepatocytes resulting in cell proliferation and liver regeneration after I/R injury.

There are no known relationships between chemokine receptors and exosome production and release. The discovery of murine homologues to human CXCR1 and CXCR2 and the subsequent generation of knockout mice for these receptors has allowed direct investigation of their roles in various pathologies [19–21]. Our previous work demonstrates that the hepatocyte chemokine receptors, CXCR1 and CXCR2, differentially regulate the regenerative response after I/R injury [10–12]. Similarly, we have shown that exosomes produced by hepatocytes promote liver regeneration after I/R injury [13]. As such, in the current study we sought to determine if there is a direct relationship between the chemokine receptors, CXCR1 and CXCR2, and exosome release in the context of regulation of liver recovery and regeneration after I/R injury.

## Materials and Methods

### Animals

Male wild type (BALB/c and C57Bl/6J), CXCR2^-/-^ mice on a BALB/c background, and CXCR1 ^-/-^ mice on a C57Bl/6J background were purchased from the Jackson Laboratory (Bar Harbor, ME). Mice used for experiments were 6–8 weeks of age. All animal use and procedures described were reviewed and approved by the University of Cincinnati Animal Care and Use Committee and was in compliance with the National Institutes of Health guidelines. Mice were group housed in individually ventilated cages with corn cob bedding and provided nestlets for environmental enrichment in rooms with a 14/10 light/dark cycle. Mice were fed irradiated standard chow (Envigo, Indianapolis, IN), and monitored daily. None of the animals became ill or died prior to the experimental endpoints. Mice were euthanized by overdose of sodium pentobarbital (10 mg/ml), followed by thoracotomy. All animals and resulting samples were assigned a number that did not reveal the group allocation so that analyses were performed by blinded investigators.

### Liver cells

Hepatocytes and Kupffer cells were isolated from mice as previously described [22]. To determine cell production of exosomes, hepatocytes or Kupffer cells were distributed onto 50-mm dishes at a concentration of 2×10^6^ cells/5 mL per dish and incubated overnight to allow cell adherence. The cells were re-incubated for 48 hours and the culture media was collected. In some experiments with isolated hepatocytes, cells were treated with 0, 100, or 5000 ng/ml macrophage inflammatory protein-2 (MIP-2), or 0–10 μM GW4869 and culture media collected 48 hours later for exosome analysis. Primary liver sinusoidal endothelial cells from C57Bl/6 mice were purchased from Cell Biologics (Chicago, IL). Cells were isolated from liver sinusoidal tissue of pathogen-free laboratory mice, and seeded at a density of 1 × 10^6^ cells/mL. Culture media was renewed on the following day and on alternating days thereafter.

### Exosome isolation and quantitation

Exosomes were purified from culture media or serum. We have previously characterized our isolated exosomes biochemically and biophysically [13]. Exosomes were isolated using differential centrifugation as previously described [23, 24]. To isolate exosomes from cultured cells, the cells were incubated in Williams media supplemented with 5% exosome-removed fetal bovine serum (System Biosciences, Mountain View, CA) for 48 hours, the culture media was collected and centrifuged at 300xg for 10 min. The supernatant was collected and centrifuged at 16,500xg for 30 min. The supernatant was then passed through a 0.22 μm filter and exosomes were harvested by 2-times centrifugation at 120,000x*g* for 70 min. The pellet was resuspended in phosphate buffered saline (PBS) and layered on a density cushion composed of Tris/sucrose/D2O solution. The samples were centrifuged at 100,000x*g* for 90 min in a SW 56 swinging bucket rotor. Then the exosomes were transferred to a fresh ultracentrifuge tube and were harvested by centrifugation at 120,000x*g* for 70 min. The final pellet was resuspended in 100 μl PBS. Serum exosomes were isolated according to the manufacturer’s protocol (System Biosciences). Briefly, blood was obtained by cardiac puncture and 125 μL of serum was collected and mixed with Exoquick exosome precipitation (System Biosciences). Samples were centrifuged at 1500xg for 30 min, followed by incubation overnight at 4°C. The supernatant was decanted and the exosome pellet was resuspended in PBS.

The size of exosomes was determined using a Zetasizer Nano (Malvern Instruments, Malvern, UK) and the number of exosomes was assessed by CD81-antigen ELISA kit (System Biosciences).

### Hepatocyte proliferation

Hepatocyte proliferation in vitro was determined by DNA incorporation of 5-bromo-2-deoxyuridine (BrdU). Hepatocytes were treated with hepatocyte-derived exosomes for 24 hours prior to assessing BrdU incorporation. Data were normalized by the amount of viable cells and expressed as a ratio compared with medium alone. A commercial BrdU cell proliferation ELISA system (Abcam, Cambridge, UK) was used for this assay.

### Hepatic I/R injury

Mice were randomly assigned to undergo either sham surgery or I/R as previously described [25]. Sham control mice underwent the same procedure without vascular occlusion. Ischemia time was 90 minutes. Serum and liver tissues were obtained prior to initiation of ischemia and 24 or 96 hours after reperfusion. Measurements of serum alanine aminotransferase (ALT) were made using a diagnostic bioassay (Wiener Laboratories, Rosario, Argentina). Liver tissues were fixed in 10% neutral-buffered formalin and embedded in paraffin. Sections were stained with hematoxylin and eosin for histologic examination.

### Ceramide staining

Hepatocytes were permeabilized by a 5min incubation with 0.05% Triton X-100 (Sigma, St. Louis, MO). Samples were washed and stained with anti-ceramide antibody (1:100 dilution, Glycobiotech, Borstel, Germany) at 4°C for 45min, followed by staining with Cy3-coupled antibody to mouse IgM (all secondary antibodies from Jackson ImmunoResearch, West Grove, PA). Ceramide staining was detected by fluorescence microscopy.

### Measurement of sphingolipid substrates and enzymes

Ceramide was quantified by kinase assays exactly as previously described [26].

Quantification of S1P in hepatocytes was determined by ELISA and mass spectrometry. An S1P ELISA (Echelon Biosciences, Salt Lake City, UT) was performed according to the manufacturer’s instructions. For mass spectrometry, S1P was extracted by a modified two-step lipid extraction. Briefly, cells were transferred into a glass tube and resuspended in 1 mL of medium. Then, 100 pmol C17-S1P as internal standard, 100 μL of a 3 N NaOH solution, 1 mL of chloroform and 1 mL of methanol/HCl (99.8:0.2 v/v) were added. After separation, the aqueous phase was acidified with 100 μL concentrated HCl and extracted with 1.5 mL chloroform. The organic phase was evaporated and the dried lipids were resolved in 200 μL methanol. Sample analysis was performed by rapid resolution liquid chromatography/tandem mass spectrometry (LC-MS/MS) using a quadrupole/time-of flight (QTOF) 6530 mass spectrometer (Agilent Technologies, Waldbronn, Germany) operating in the positive electrospray ionization (ESI) mode. Chromatographic separations were performed by a X-Bridge column (C18, 4.6x150 mm, 3.5 μm particle size, 138 Å pore size, Waters GmbH, Eschborn, Germany). Elution was performed using a gradient consisting of eluent A (water/formic acid 100:0.1 v/v) and eluent B (acetonitril/tetrahydrofuran/formic acid 50:50:0.1 v/v/v). The precursor ions of S1P (m/z 380.2560) and C17-S1P (m/z 366.2404) were cleaved into the fragment ions of m/z 264.2700 and m/z 250.2529 respectively. Quantification was performed with Mass Hunter Software.

Neutral sphingomyelinase (Nsm) activity was measured by incubation of samples with 0.05 μCi per sample [^14^C]sphingomyelin in 100 mM Tris-HCl (pH 7.4), 5 mM MgCl_2_, 2.5 mM DTT, 0.2% Triton, 10 μg/ml each of aprotinin and leupeptin for 60 min at 37°C. The [^14^C]sphingomyelin was dried prior to analysis, resuspended in the assay buffer, sonicated for 10 min and an aliquot was added to the samples. The reactions were terminated by addition of 1 mL CHCl_3_:CH_3_OH (2:1, v/v), samples were centrifuged for 5 min at 14 000 rpm, an aliquot of the upper phase was removed and liquid scintillation counted to calculate Nsm activity.

Neutral ceramidase activity was measured by incubating samples in 100 mM Tris-HCl (pH 7.4), 5 mM MgCl_2_, 2.5 mM DTT, 0.2% Triton, 10 μg/ml each of aprotinin and leupeptin and 0.1 μCi micellar [^14^C16]-ceramide (ARC0831, 55 mCi/mmol). The substrate was dried prior to use, resuspended in the assay buffer and bath-sonicated for 10 min. Samples were extracted after 60 min in 200 μl H_2_O and CHCl_3_:CH_3_OH:HCl (100:100:1, v/v/v). The lower phase was dried, samples were resuspended in CHCl_3_:CH_3_OH (1:1, v/v) and separated by TLC using CHCl_3_:CH_3_OH:ammoniumhydroxide (90:20:0.5, v/v/v) as the developing solvent. The plates were analyzed using a Fuji-Imager and ceramidase activity was determined by conversion of radioactive ceramide into sphingosine and radioactive fatty acid.

Sphingosine kinase activity was measured by incubation of samples with 500 pmol sphingosine in the presence of 50 mM HEPES (pH 7.4), 250 mM NaCl, 30 mM MgCl_2_, 1 mM ATP and 10 μCi [^32^P]γATP for 60 min at 30°C. Samples were extracted by addition of 20 μl 1N HCl, 800 μl CHCl_3_/CH_3_OH/1N HCl (100:200:1, v/v/v), 240 μl CHCl_3_ and 2 M KCl. Phases were separated, the lower phase was collected, dried, dissolved in 20 μL of CHCl_3_:CH_3_OH (1:1, v/v) and separated on Silica G60 thin-layer chromatography (TLC) plates using CHCl_3_/CH_3_OH/acetic acid/H_2_O (90:90:15:5, v/v/v/v). The TLC plates were analyzed employing a phosphoimager. Sphingosine was quantified by comparison with a standard curve of C18-sphingosine and sphingosine kinase activity was calculated from the conversion of the standards to S1[^32^P].

### Rab27a/b protein expression

Expression of Rab27a, Rab27b, and β-actin were determined by Western blot. Isolated murine hepatocytes were resuspended with RIPA buffer and cellular debris was removed by centrifugation at 10,000 rpm. Protein concentrations of each sample was determined using a BCA protein assay kit (Pierce/ThermoFisher, Rockford, IL). Samples containing equal amounts of protein were resuspended in 5x SDS sample buffer and separated by 4–20% SDS-PAGE and transferred to a 0.1μm pore polyvinylidene fluoride membrane. Nonspecific binding sites were blocked with tris-buffered saline (40 mM Tris, pH 7.6, 300mM NaCl) with 0.1% Tween 20 containing 5% non-fat dry milk for 1 hour at room temperature. Membranes were then incubated with antibodies to anti-Rab27a (ab55667, Abcam Inc., Cambridge, MA), anti-Rab27b (ABS1026, EMD Millipore, Darmstadt, Germany), or anti-β-actin (ab8227, Abcam Inc.) in tris-buffered saline with 0.1% Tween 20. Membranes were washed and incubated with secondary antibodies conjugated to horseradish peroxidase. Immunoreactive proteins were detected by enhanced chemiluminescence.

### Statistical analysis

Sample size was calculated with the two-sided Wilcoxon-Mann-Whitney test (software: G*Power Version 3.1.7). Data are expressed as the mean ± standard error of the mean (SEM). Data were analyzed with a one-way analysis of variance, with a subsequent Student t-test. Differences were considered significant when *P* < 0.05.

## Results

### Exosome release from hepatocytes is dependent upon CXCR1 and CXCR2

**Fig 1:**
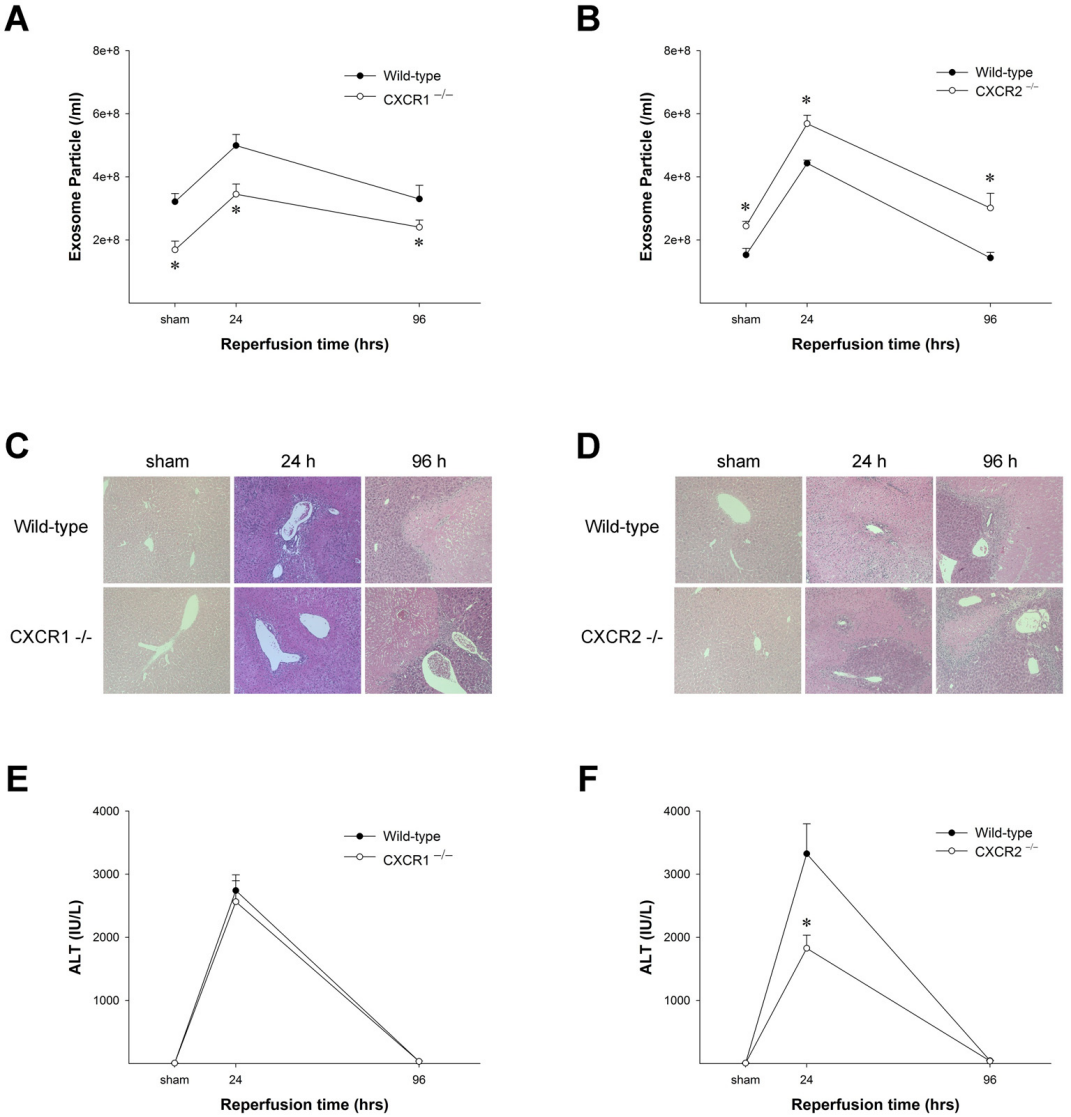
CXCR1 and CXCR2 regulate exosome release during hepatic I/R injury. Knockout of CXCR1 (A) reduced, while knockout of CXCR2 (B) increased the number of exosomes in the serum during ischemia/reperfusion (I/R) injury. After 24 and 96 hours of reperfusion, serum was taken for analysis. Serum exosomes were determined via CD81 antigen ELISA. Data are mean ± SEM with n = 4–11 per group. *P<0.05 compared to wild-type mice. Liver histology in CXCR1-knockout mice was similar to wild-types (C), whereas CXCR2-knockout mice showed improve liver architecture after I/R injury (D). Similarly, serum ALT values in CXCR1-knockout mice were similar to wild-type controls (E), while CXCR2-knockout mice had significantly less ALT than their wild-type controls (F). Data are mean ± SEM with n = 3–4 per group. *P<0.05 compared to wild-type mice.

Our previous work demonstrated that CXCR1 and CXCR2 function in opposing ways during repair and regeneration after I/R injury. Mice lacking CXCR1 show delayed liver regeneration after I/R injury, while mice lacking CXCR2 had accelerated regeneration [11, 12]. To determine if these responses might be related to the release of exosomes by hepatocytes, we measured the number of exosomes in serum before and after hepatic I/R injury in wild-type and CXCR1- or CXCR2-knockout mice. Similar to our previous study, exosomes were present in the serum of untreated mice and increased after I/R with maximum levels 24 hours after reperfusion and normalization to baseline levels 96 hours after reperfusion (Fig 1A and 1B). Interestingly, serum from CXCR1-knockout mice showed fewer exosomes (Fig 1A), whereas serum from CXCR2-knockout mice showed a significantly higher number of exosomes compared to wild-type controls (Fig 1B). Consistent with our earlier studies, histological analysis of liver sections demonstrated that CXCR1-knockout mice had severe liver injury that was similar to their wild-type counterparts (Fig 1C), whereas CXCR2-knockout mice had far less liver injury 96 hours after reperfusion (Fig 1D). Similarly, there were no differences in serum ALT levels in CXCR1-knockout mice versus wild-types (Fig 1E), but CXCR2-knockout mice had significantly lower ALT levels 24 hours after reperfusion compared to wild-type controls (Fig 1F).

**Fig 2:**
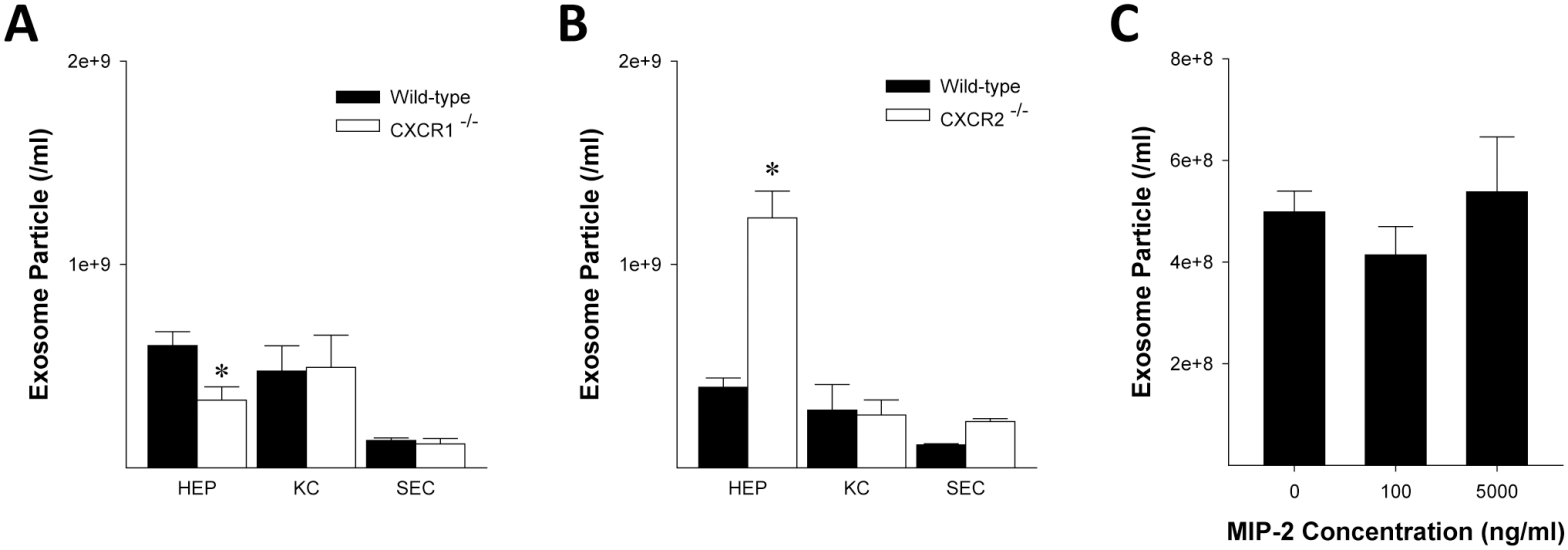
CXCR1 and CXCR2 regulate exosome release in hepatocytes independent of ligand. Exosomes released by wild-type and CXCR1-deficient (A) or CXCR2-deficient (B) hepatocytes (HEP), Kupffer cells (KC), and liver sinusoidal endothelial cells (SEC) were measured by CD81 antigen ELISA. Data are mean ± SEM with n = 3–6 per group. *P<0.05 compared to wild-type cells. (C) Wild-type hepatocytes were treated with 0, 100, or 5000 ng/ml MIP-2 and exosomes in the culture media were determined via CD81 ELISA. Data are mean ± SEM with n = 7–13 per group.

To determine what cell type(s) were responsible for these differences in serum exosome numbers, we assessed the number of exosomes released by isolated hepatocytes, Kupffer cells, and liver sinusoidal endothelial cells, all which are known to be primary regulators of liver recovery and regeneration after I/R. Interestingly, CXCR1-knockout hepatocytes produced fewer exosomes (Fig 2A), whereas CXCR2-knockout hepatocytes produced more exosomes compared to their wild-type controls (Fig 2B). We found no differences in the number of exosomes released by Kupffer cells or liver sinusoidal endothelial cells from wild-type or chemokine receptor knockout mice (Fig 2A and 2B). We next tested if chemokine ligands alter the release of exosomes by hepatocytes. Treatment of wild-type hepatocytes with a wide dose range of the CXC chemokine, MIP-2, had no effect on exosome release (Fig 2C). These data suggest that the expression of CXCR1 and CXCR2 directly regulates the production and/or release of exosomes selectively in hepatocytes and that this regulation is independent of ligand/receptor interactions.

### CXCR2, but not CXCR1, modulates neutral sphingomyelinase (Nsm) activity to control exosome release by hepatocytes

**Fig 3:**
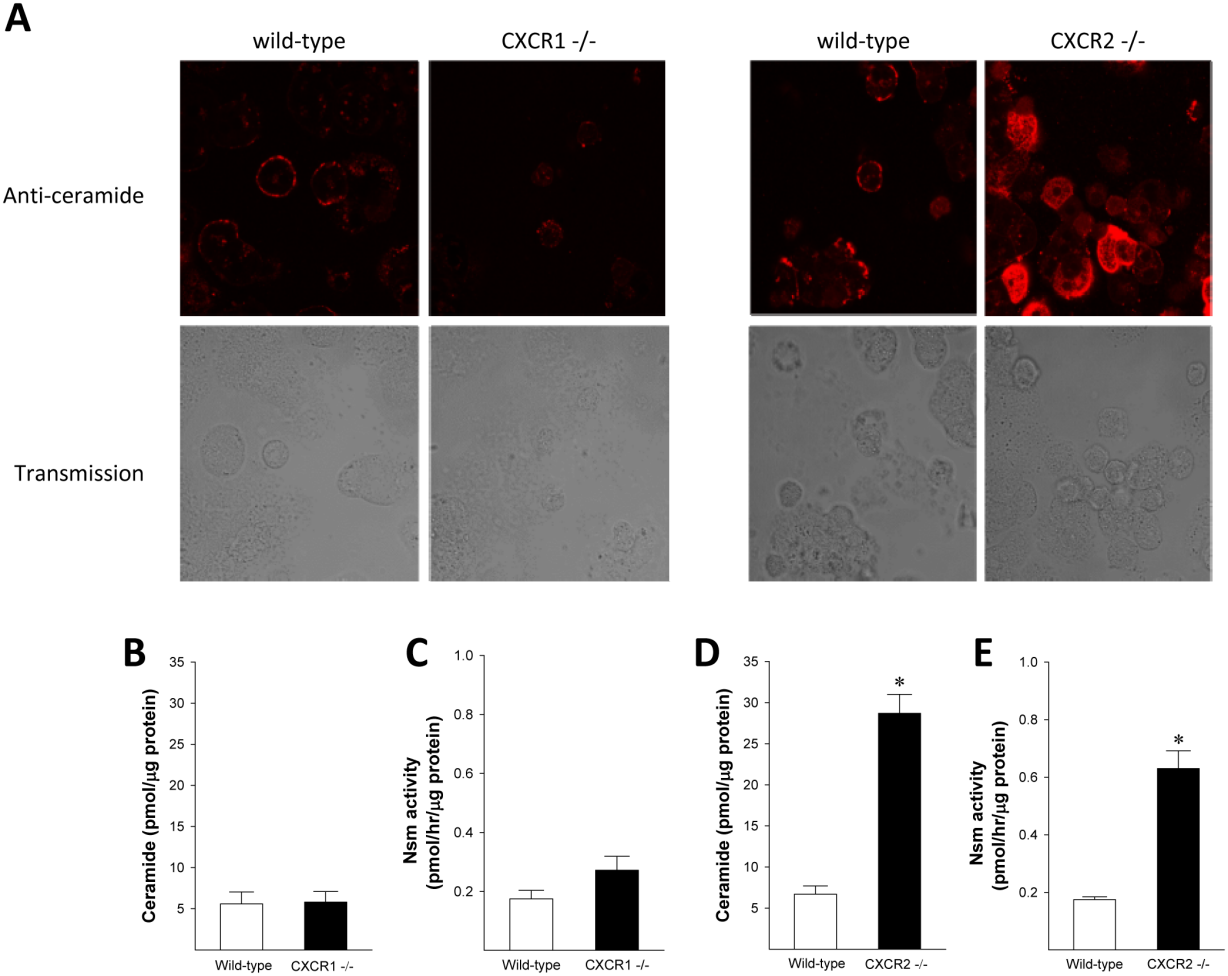
CXCR2, but not CXCR1, modulates Nsm and ceramide to regulate exosome release. Accumulated ceramide in wild-type and CXCR1-deficient or CXCR2-deficient hepatocytes was detected fluorescence microscopy with Cy-3-labeled antibody (A). Intracellular hepatocyte ceramide concentrations (B, D) and Nsm activity (C, E) were determined. Data are mean ± SEM with n = 4 per group. *P<0.05 compared to wild-type mice. *P<0.05 compared to wild-type mice.

**Fig 4:**
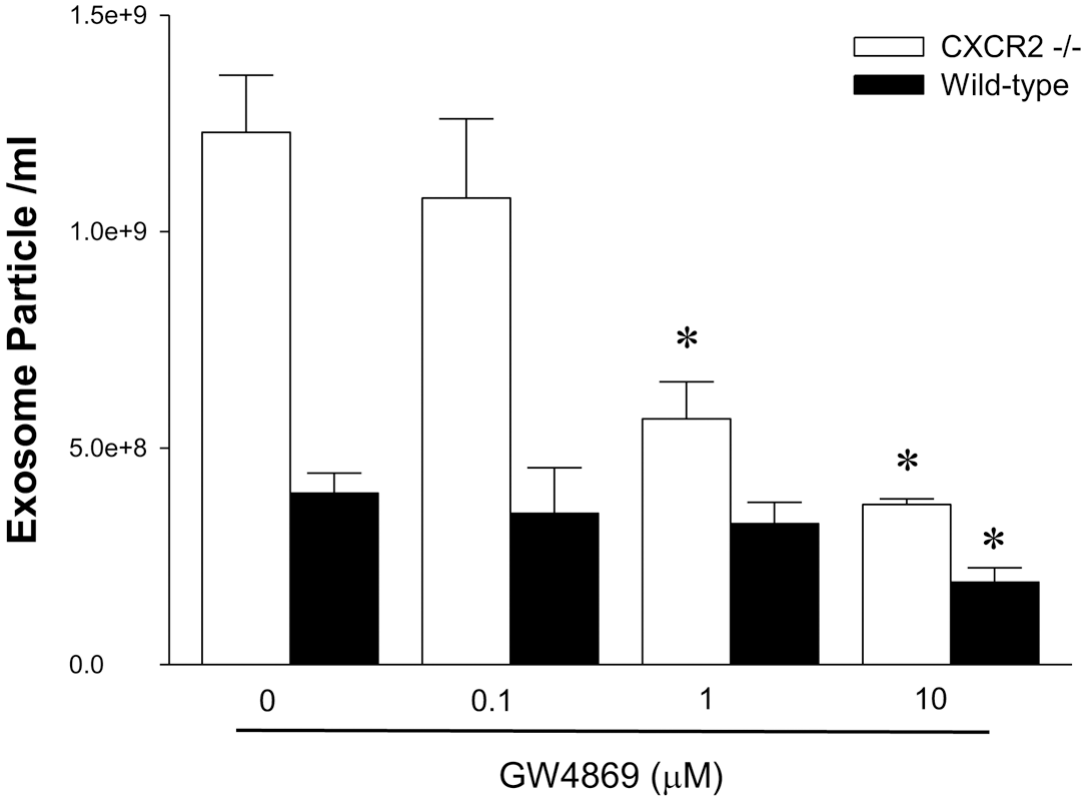
Inhibition of Nsm reduces exosome release in CXCR2-deficient hepatocytes. Exosomes released by wild-type and CXCR2-deficient hepatocytes treated with the Nsm inhibitor, GW4869, were measured by CD81 antigen ELISA. Data are mean ± SEM with n = 4–8 per group. *P<0.05 compared to control group (0 μM GW4869).

Ceramide is generated in cell membranes from sphingomyelin by Nsm [27, 28]. Other studies have shown that ceramide and Nsm are key regulators of exosome formation [29]. To determine whether the altered exosome release observed in CXCR1- or CXCR2-deficient hepatocytes was related to alterations in the sphingomyelinase-ceramide pathway, we measured ceramide levels in hepatocytes from wild-type or knockout mice. Isolated hepatocytes were stained with an anti-ceramide antibody. CXCR1-deficient hepatocytes showed similar levels of ceramide expression (Fig 3A), whereas CXCR2-deficient hepatocytes showed markedly increased ceramide expression compared to wild-type controls (Fig 3A). To verify these findings, we measured the concentration of ceramide and Nsm activity in isolated hepatocytes by mass spectroscopy, kinase assay and enzymatic assay, respectively. Consistent with our ceramide staining data, ceramide concentrations in CXCR1-deficient hepatocytes were similar to their wild-type controls (Fig 3B), whereas CXCR2-deficient hepatocytes had a ceramide concentration approximately 5x higher than wild-type controls (Fig 3D). Similarly, Nsm activity was not different in CXCR1-deficient hepatocytes (Fig 3C), but CXCR2-deficient hepatocytes had markedly increased Nsm activity compared to wild-type controls (Fig 3E). Treatment of CXCR2-deficient hepatocytes with the neutral sphingomyelinase inhibitor, GW4869, dose-dependently reduced the number of exosomes released and at 10 μM of GW4869 exosome release was equivalent to wild-type hepatocytes (Fig 4). These data suggest that hepatocyte expression of CXCR2, but not CXCR1, has a regulatory effect on Nsm that controls intracellular ceramide levels and subsequent production and release of exosomes.

### CXCR1 and CXCR2 do not regulate Rab27 proteins or exosome size

**Fig 5:**
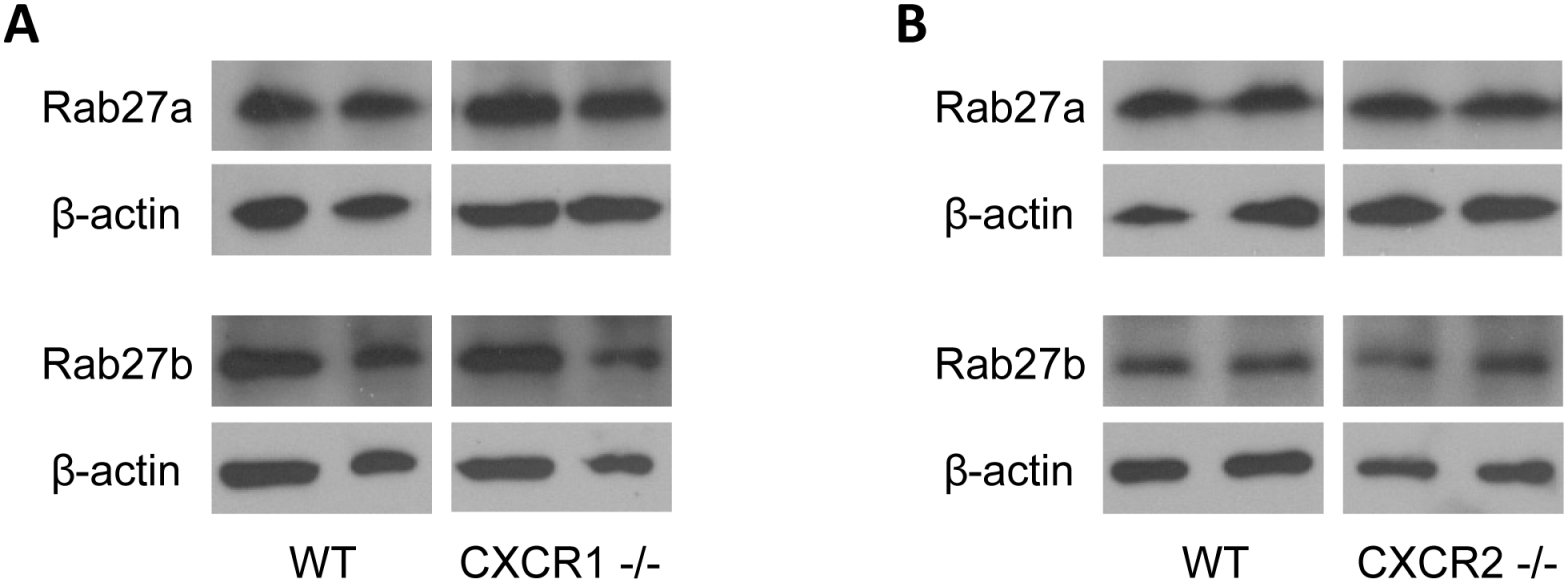
Rab27 proteins are not regulated by CXCR1 or CXCR2. Protein expression of Rab27a and Rab27b in wild-type and CXCR1-deficient (A) or CXCR2-deficient (B) hepatocytes was determined by Western blot. β-actin was used as a control protein. Results shown are representative of three independent experiments.

Two Rab GTPases, Rab27a and Rab27b, have been shown to regulate exosome release by facilitating intracellular trafficking of multivesicular endosomes and their docking at the plasma membrane [30]. We next explored if the changes in exosome release in CXCR1- or CXCR2-deficient hepatocytes was related to altered expression of these proteins. We observed no differences in the expression of Rab27a and Rab27b in CXCR1-deficient (Fig 5A) or CXCR2-deficient (Fig 5B) hepatocytes compared to wild-type controls. Because Rab27 proteins are also known to regulate exosome size [27], we next assessed whether knockout of CXCR1 or CXCR2 altered the size of exosomes released by hepatocytes. Quantitation of exosome size was determined using a Zetasizer Nano and revealed no differences in mean vesicle diameters of exosomes released from CXCR1- or CXCR2-deficient hepatocytes compared to their wild-type controls (S1 Fig).

### CXCR1 regulates the contents of hepatocyte exosomes that promote hepatocyte proliferation

**Fig 6:**
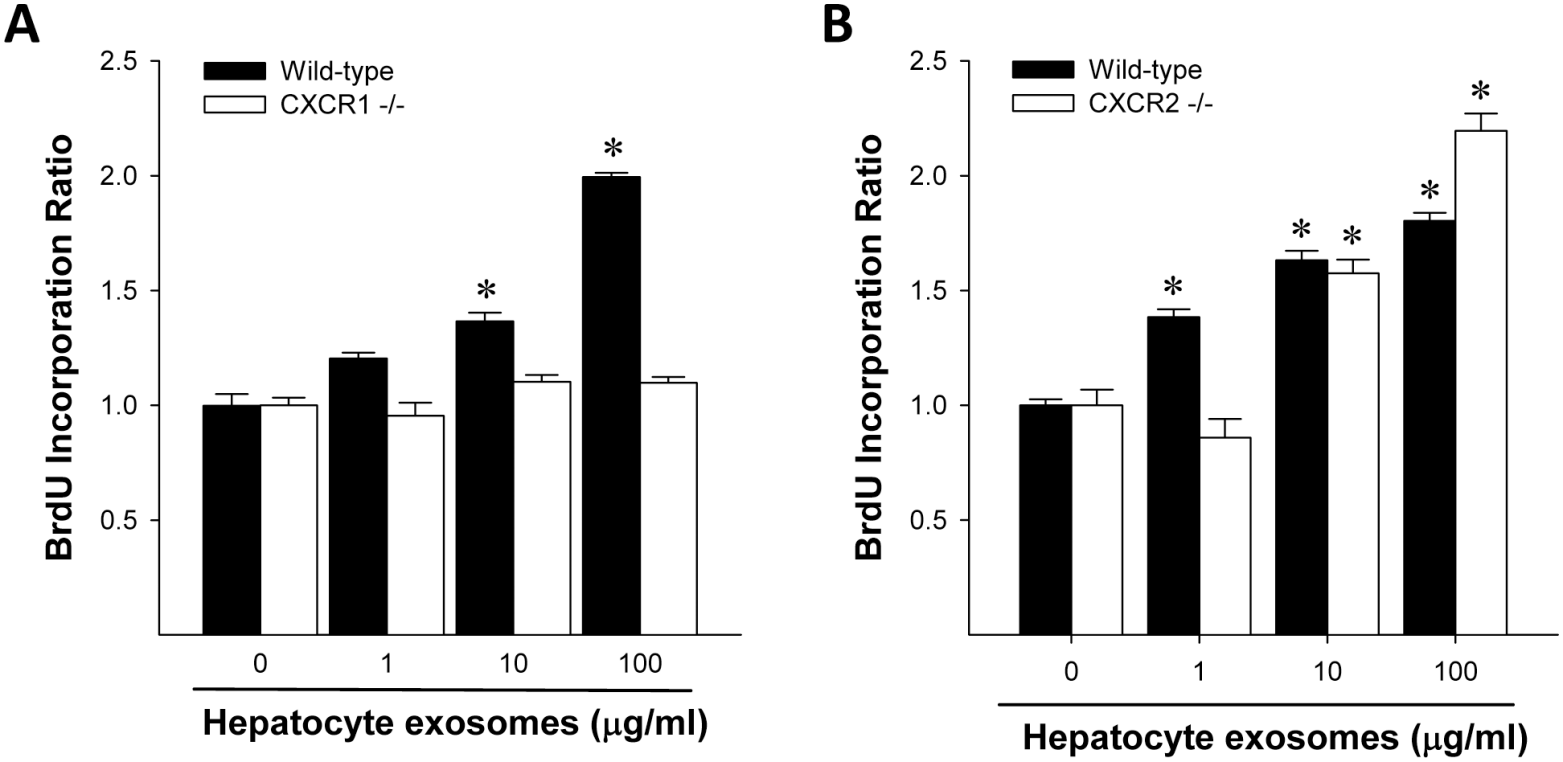
Knockout of CXCR1 abrogates the proliferative effects of hepatocyte exosomes. Primary hepatocytes from wild-type mice were treated with exosomes from wild-type and CXCR1-deficient (A) or CXCR2-deficient (B) hepatocytes for 24 hours. Cell proliferation was determined by BrdU incorporation assays. Data are mean ± SEM with n = 8 per group. *P<0.05 compared to control group (0 μg/ml exosomes).

We next sought to determine if CXCR1 or CXCR2 regulated the ability of hepatocyte exosomes to promote hepatocyte proliferation. Similar to our previous study, exosomes from wild-type hepatocytes induced dose-dependent increases in hepatocyte proliferation in vitro (Fig 6A and 6B). Interestingly, exosomes from CXCR1-deficient hepatocytes had no effect on hepatocyte proliferation (Fig 6A), whereas exosomes from CXCR2-deficient hepatocytes were similar to those from wild-type hepatocytes (Fig 6B).

**Fig 7:**
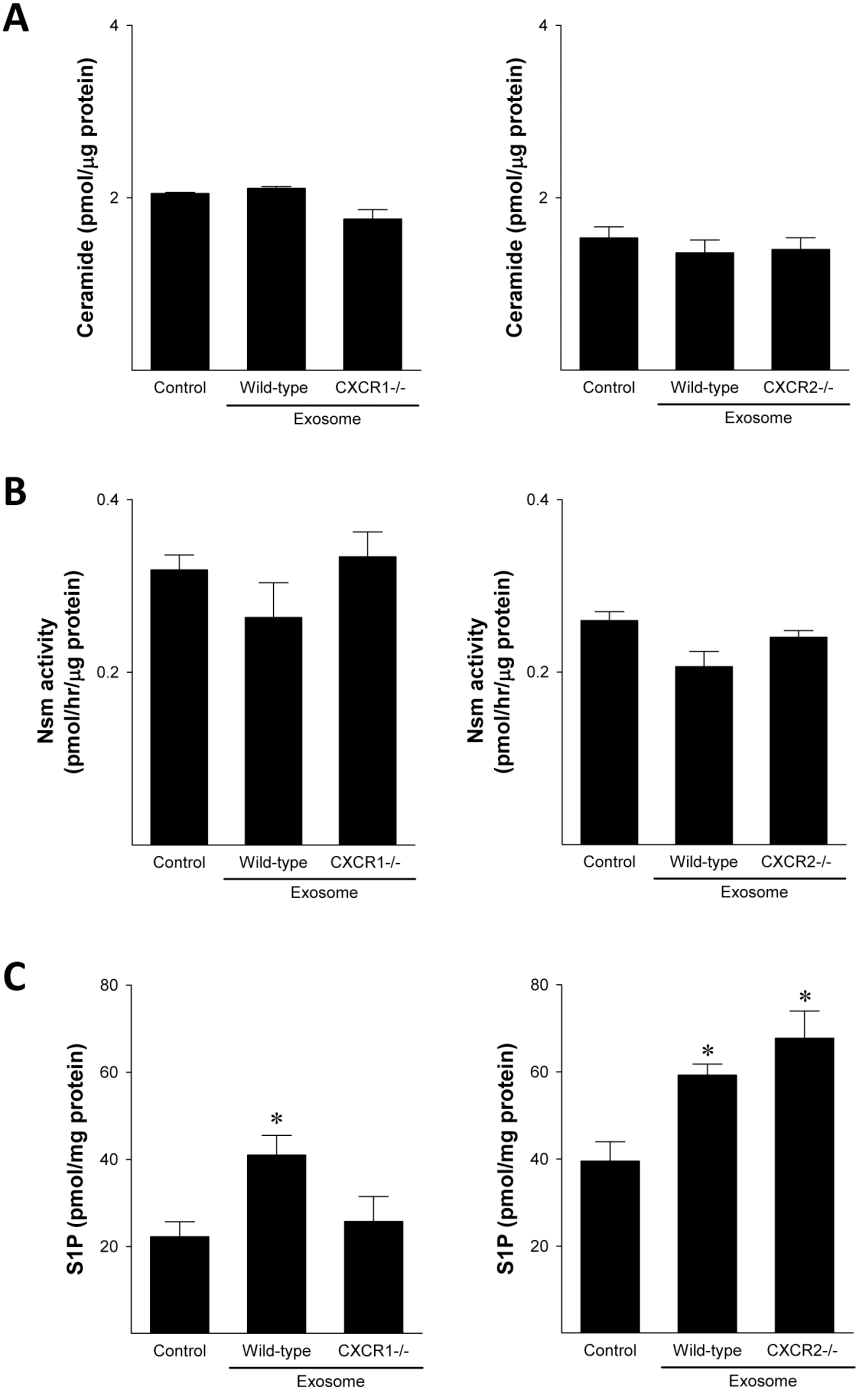
Exosomes from CXCR1-deficient hepatocytes do not induce sphingosine-1-phosphate (S1P) generation in target hepatocytes. Primary hepatocytes were treated with media (control) or 100 μg/ml hepatocyte exosomes and cellular ceramide concentration (A), neutral sphingomyelinase (Nsm) activity (B) and S1P (C) were determined. Data are mean ± SEM with n = 4 per group. *P<0.05 compared to control group.

Because we have previously demonstrated that hepatocyte exosomes promote hepatocyte proliferation and liver regeneration by delivering sphingosine kinase 2 to target hepatocytes resulting in increased intracellular S1P [13], we next assessed intracellular changes in hepatocytes treated with exosomes. Treatment with exosomes from wild-type or chemokine receptor deficient hepatocytes had no effect on intracellular concentration of ceramide or activity levels of intracellular Nsm (Fig 7A and 7B). Consistent with our previous work showing that hepatocyte exosomes induce an increase in intracellular S1P generation [13], we found that exosomes from wild-type hepatocytes significantly increased S1P in target hepatocytes (Fig 7C). Interestingly, exosomes from CXCR1-deficient hepatocytes induced no increase in S1P production, whereas exosomes from CXCR2-deficient hepatocytes were similar to those from wild-type hepatocytes (Fig 7C).

**Fig 8:**
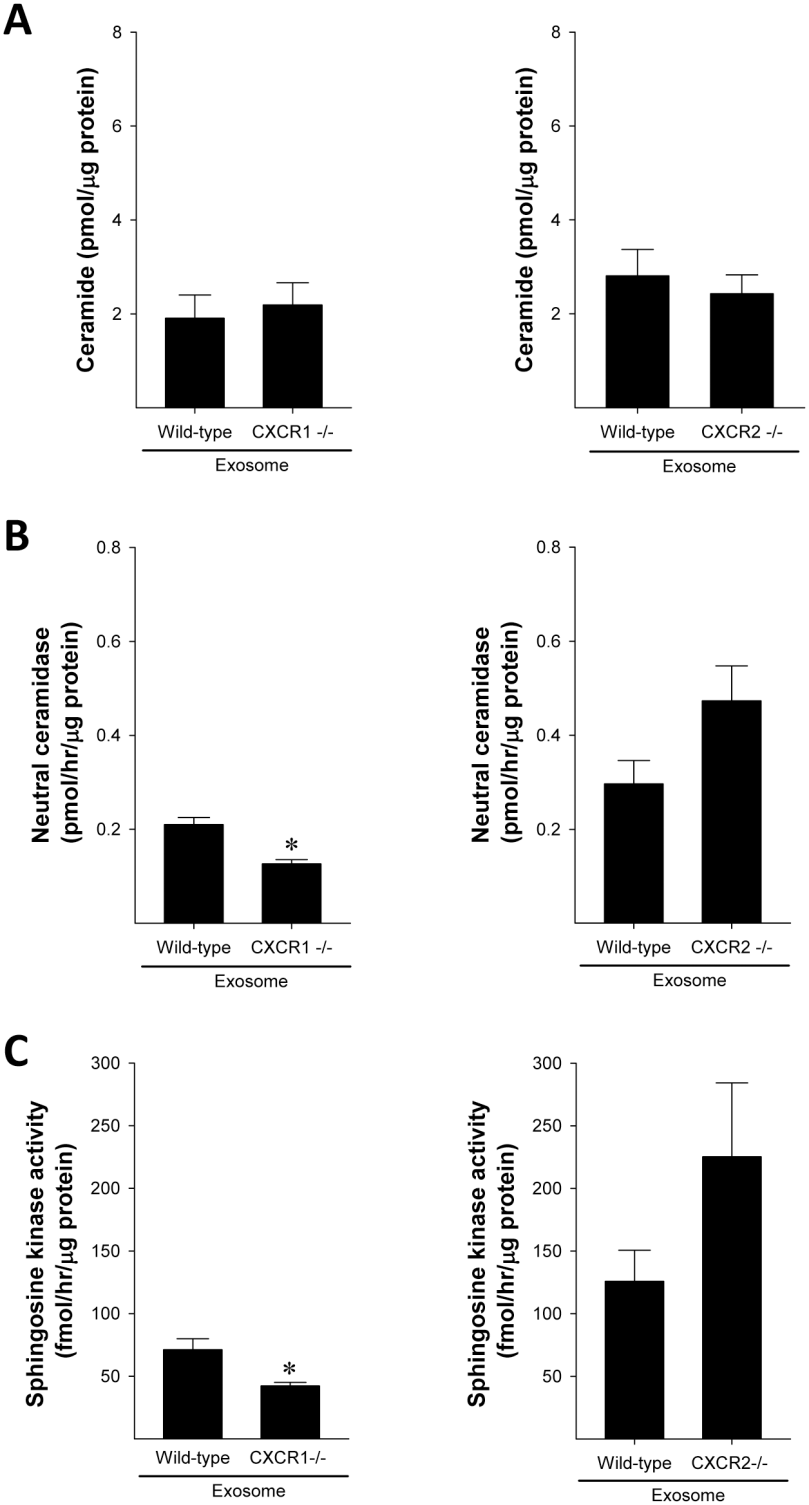
Exosomes from CXCR1-deficient hepatocytes lack neutral ceramidase and sphingosine kinase activity. Ceramide concentration (A), neutral ceramidase activity (B), and sphingosine kinase activity (C) were measured in exosomes from wild-type and CXCR1- and CXCR2-deficient hepatocytes. Data are mean ± SEM with n = 3 per group. *P<0.05 compared to wild-type exosomes.

To determine if the lack of intracellular S1P generation and subsequent proliferation in hepatocytes treated with exosomes from CXCR1-deficient hepatocytes might be due to differences in exosome contents, we next measured the concentration of ceramide and activity of neutral ceramidase and sphingosine kinase 2 in exosomes. No differences were observed in ceramide concentrations between exosomes from wild-type hepatocytes and chemokine-deficient hepatocytes (Fig 8A). However, exosomes from CXCR1-deficient hepatocytes had significantly less neutral ceramidase activity (Fig 8B) and sphingosine kinase activity (Fig 8C) compared to exosomes from wild-type hepatocytes. Exosomes from CXCR2-deficient hepatocytes showed trends of increased activity of neutral ceramidase and sphingosine kinase, but these were not significantly different from wild-type controls (Fig 8B and 8C).

## Discussion

To the best of our knowledge, the current study is the first to discover a direct relationship between CXC chemokine receptor expression and release of exosomes by hepatocytes. Our data demonstrate that hepatocytes that lack the receptor, CXCR1, produce less exosomes than their wild-type counterparts. In contrast, hepatocytes that lack expression of CXCR2 produce significantly more exosomes than wild-type cells. It is important to note that the regulation of hepatocyte exosome release by these receptors is unrelated to ligand binding. This is supported by two findings. First, that receptor knockout mice had altered numbers of circulating exosomes in the absence of I/R injury, and second, that the addition of chemokine ligands to isolated hepatocytes had no effect on exosome release. This may suggest new regulatory mechanisms of exosome release in hepatocytes that may occur in intracellular compartments.

Our work defines a novel role for the chemokine receptor, CXCR2, in regulating hepatocyte exosome formation. While we clearly show that CXCR2 regulates exosome production and release by modulating the activity of Nsm and resultant production of ceramide, the precise molecular interactions between CXCR2 and Nsm remains to be determined. CXCR2 is most often thought of in the context of a cell surface receptor that binds to a variety of ligands and initiates a signal transduction cascade via G-proteins [31]. However, Nsm is localized to the cytoplasmic leaflet of plasma and organelle membranes [29]. Since CXCR2 can be internalized via endocytosis [32], it is possible that CXCR2 and Nsm directly interact in endosomal membranes or in membranes of multivesicular bodies that ultimately generate exosomes. The nature of such an interaction would represent a completely novel function of chemokine receptors.

Our data do not support such a mechanism for the regulatory effects of CXCR1 on hepatocyte exosome release. We found no changes in neutral sphingomyelinase activity, intracellular ceramide concentration or expression of Rab27a or Rab27b. The latter two proteins have been shown to regulate the pathways of exosome release [30]. While our efforts were unable to delineate the precise mechanism by which CXCR1 regulate exosome production and/or release, we did find that CXCR1 is critical for the packaging of neutral ceramidase and sphingosine kinase 2 into exosomes. We have shown previously that these enzymes, specifically sphingosine kinase 2, are critical for the proliferative effects of hepatocyte exosomes [13]. In our current work, we demonstrate that exosomes from CXCR1-deficient hepatoctyes are completely devoid of the proliferative effects observed in exosomes from wild-type hepatoctyes. Whether the regulatory effects of CXCR1 on exosome cargo may be related to the numbers of exosomes produced or released by hepatocytes is unclear.

Collectively, our work over the past several years suggests there are multiple mechanisms by which the CXC chemokine receptors, CXCR1 and CXCR2, regulate the reparative and regenerative responses of the liver after injury. Both receptors promote hepatocyte proliferation upon ligand binding, but high concentrations of ligand may also induce signaling pathways resulting in cell death [11, 12, 33]. In the present work, we find completely different functions of CXCR1 and CXCR2 in the regulation of the production and/or release of exosomes by hepatocytes and, in the case of CXCR1, a role in protein packaging into these exosomes. The fact that we found decreased numbers of exosomes in CXCR1 knockout mice is consistent with our previous finding that these mice have a delayed reparative/regenerative response after I/R injury [11]. Likewise, CXCR2-knockout mice release much greater amounts of hepatocyte exosomes and show greatly accelerated repair and regeneration of post-ischemic liver [12]. It would seem likely that the altered release of exosomes in these mutant mice has a direct impact on the regenerative response of the liver.

In summary, the current study demonstrates that the CXC chemokine receptors, CXCR1 and CXCR2, directly influence the production and/or release of exosomes in hepatocytes. These effects are not ligand mediated and for CXCR2 are directly related to the activity of neutral sphingomyelinase and resultant generation of intracellular ceramide. The mechanism by which CXCR1 regulates exosome production or release is unclear, but CXCR1 is required for the packaging of neutral ceramidase and SK2 into exosomes. The lack of these proteins in exosomes from CXCR1-deficient hepatocytes abrogates the proliferative effects of the exosomes. These data provide novel information regarding the diverse functions of CXC chemokine receptors in hepatocyte biology and in liver repair and regeneration.

## Supporting Information

S1 Fig CXCR1 and CXCR2 do not regulate the size of exosome from hepatocyte. The diameter of exosomes released from wild-type and CXCR1- and CXCR2-deficient hepatocyte was determined using a Zetasizer Nano.
(PDF)

S1 Table Datasets for Figs 1–4 and Figs 6–8. Datasets for individual figures are in individual tabs of the spreadsheet.
(XLSX)
