# Supplementary figures and images for: Chemokine Receptors, CXCR1 and CXCR2, Differentially Regulate Exosome Release in Hepatocytes
Source: PLoS One. 2016 Aug 23;11(8):e0161443. doi: 10.1371/journal.pone.0161443 (PMC4995008; doi:10.1371/journal.pone.0161443)

# Exosome size

BALB/c hepatocyte

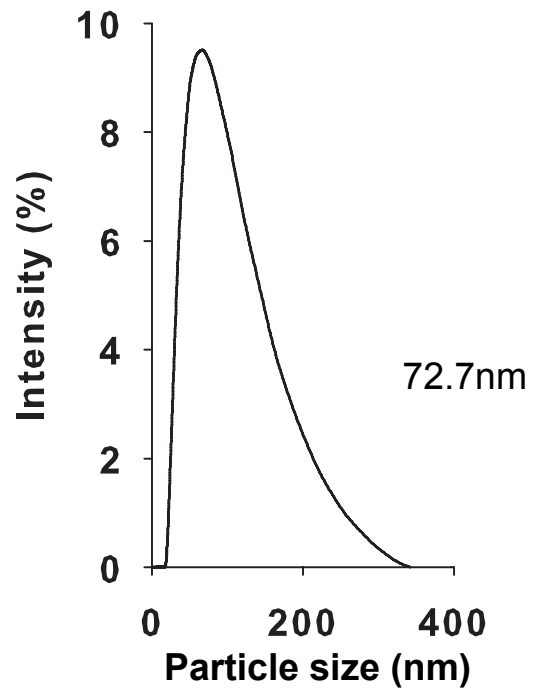

CXCR2<sup>-/-</sup> hepatocyte

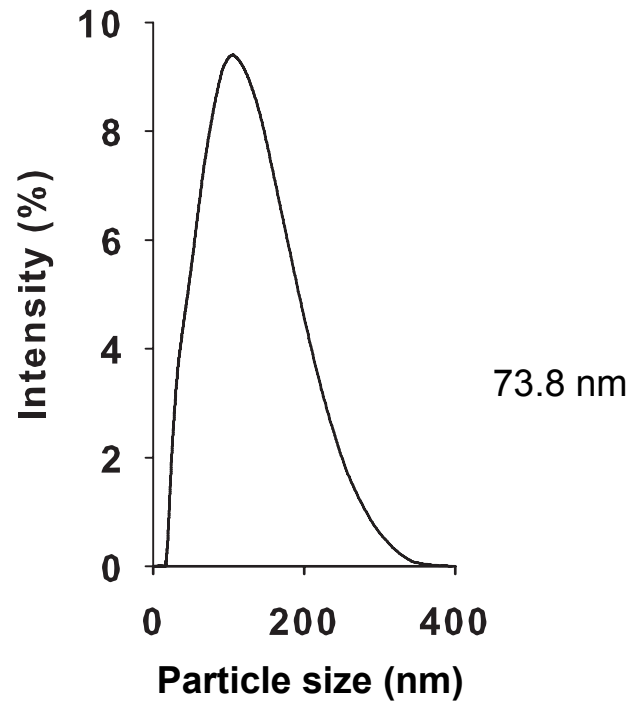

C57BL/6 hepatocyte

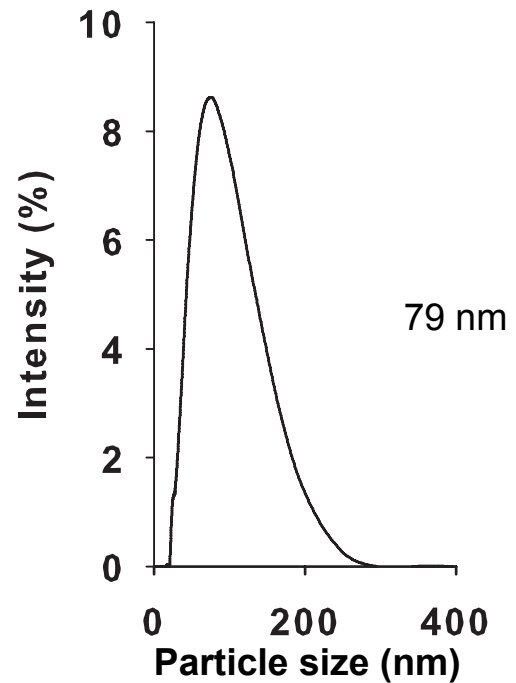

CXCR1<sup>-/-</sup> hepatocyte

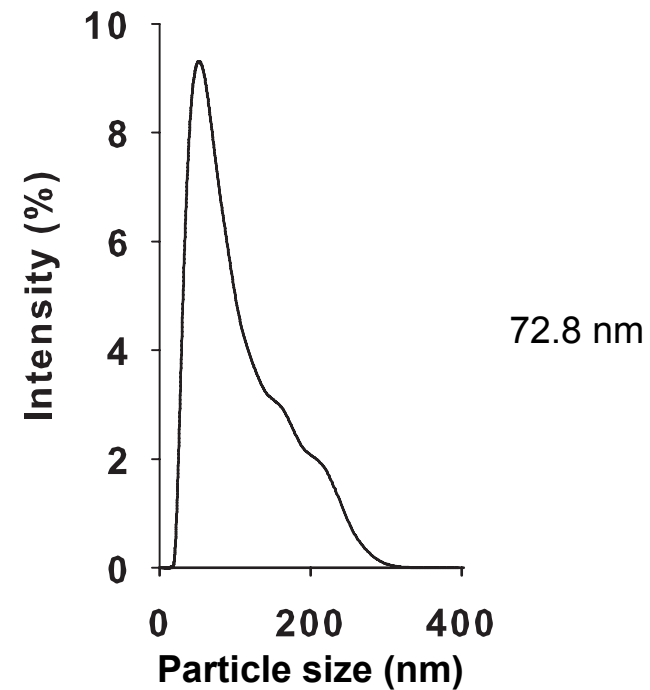

Supplement: S1 Fig — The diameter of exosomes released from wild-type and CXCR1- and CXCR2-deficient hepatocyte was determined using a Zetasizer Nano. (PDF) [file pone.0161443.s001.pdf]
